# Functional and Biochemical Characterization of Three Recombinant Human Glucose-6-Phosphate Dehydrogenase Mutants: Zacatecas, Vanua-Lava and Viangchan

**DOI:** 10.3390/ijms17050787

**Published:** 2016-05-21

**Authors:** Saúl Gómez-Manzo, Jaime Marcial-Quino, America Vanoye-Carlo, Hugo Serrano-Posada, Abigail González-Valdez, Víctor Martínez-Rosas, Beatriz Hernández-Ochoa, Edgar Sierra-Palacios, Rosa Angélica Castillo-Rodríguez, Miguel Cuevas-Cruz, Eduardo Rodríguez-Bustamante, Roberto Arreguin-Espinosa

**Affiliations:** 1 Laboratorio de Bioquímica Genética, Instituto Nacional de Pediatría, México D.F. 04530, Mexico; razer_45r@hotmail.com; 2 CONACYT—Instituto Nacional de Pediatría, México D.F. 04530, Mexico; racastilloro@conacyt.mx; 3 Laboratorio de Neurociencias, Instituto Nacional de Pediatría, México D.F. 04530, Mexico; america_vc@yahoo.com.mx; 4 CONACYT—Laboratorio de Bioingeniería, Universidad de Colima, Colima 28400, Mexico; hjserranopo@conacyt.mx; 5 Departamento de Biología Molecular y Biotecnología, Instituto de Investigaciones Biomédicas, Universidad Nacional Autónoma de México, México D.F. 04510, Mexico; abigaila@correo.biomedicas.unam.mx; 6 Laboratorio de Inmunoquímica, Hospital Infantil de México Federico Gómez, México D.F. 06720, Mexico; beatrizhb_16@comunidad.unam.mx; 7 Colegio de Ciencias y Humanidades, Plantel Casa Libertad, UACM, México D.F. 09620, Mexico; edgar.sierra@uacm.edu.mx; 8 Departamento de Química de Biomacromoléculas, Instituto de Química, Universidad Nacional Autónoma de México, Circuito Exterior s/n, Ciudad Universitaria, México D.F. 04510, Mexico; miguel.ccqi@yahoo.com.mx (M.C.-C.); e-rodriguez-bustamante@ciencias.unam.mx (E.R.-B.); arrespin@unam.mx (R.A.-E.)

**Keywords:** glucose-6-phosphate dehydrogenase (G6PD) deficiency, human G6PD mutants, steady state kinetics, thermostability, structural characterization

## Abstract

Glucose-6-phosphate dehydrogenase (G6PD) deficiency in humans causes severe disease, varying from mostly asymptomatic individuals to patients showing neonatal jaundice, acute hemolysis episodes or chronic nonspherocytic hemolytic anemia. In order to understand the effect of the mutations in *G6PD* gene function and its relation with G6PD deficiency severity, we report the construction, cloning and expression as well as the detailed kinetic and stability characterization of three purified clinical variants of G6PD that present in the Mexican population: G6PD Zacatecas (Class I), Vanua-Lava (Class II) and Viangchan (Class II). For all the G6PD mutants, we obtained low purification yield and altered kinetic parameters compared with Wild Type (WT). Our results show that the mutations, regardless of the distance from the active site where they are located, affect the catalytic properties and structural parameters and that these changes could be associated with the clinical presentation of the deficiency. Specifically, the structural characterization of the G6PD Zacatecas mutant suggests that the R257L mutation have a strong effect on the global stability of G6PD favoring an unstable active site. Using computational analysis, we offer a molecular explanation of the effects of these mutations on the active site.

## 1. Introduction

**Figure 1:**
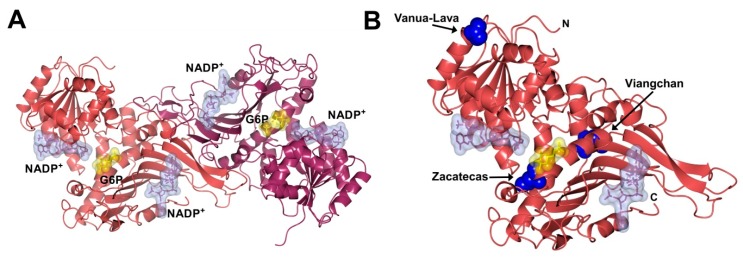
Crystallographic structure of the human glucose-6-phosphate dehydrogenase (G6PD) enzyme: (**A**) The human Wild Type (WT) structure of G6PD dimer (PDB entries 2BHL and 2BH9) showing the NADP^+^ binding (ice blue molecular surface) at the structural and coenzyme sites and the G6P site (yellow molecular surface). The two monomers are shown in pale crimson and dark purple; (**B**) Zacatecas, Viangchan and Vanua-Lava mutants are shown as blue spheres in the human G6PD structure. The graphical representations were also constructed with CCP4mg version 2.10.4 [18].

Glucose-6-phosphate dehydrogenase (G6PD) (EC 1.1.1.49) is a cytosolic enzyme [1] involved in the first step of the pentose phosphate pathway, which catalyzes the conversion of β-d-glucose-6-phosphate into d-glucono-1,5-lactone-6-phosphate with the concomitant production of one molecule of nicotinamide adenine dinucleotide phosphate (NADPH) (Figure 1A). When the 6-phosphogluconate becomes ribulose 5-phosphate by 6-phosphogluconate dehydrogenase (6PGD) enzyme, a second molecule of NADPH is produced. The G6PD enzyme plays a fundamental role in erythrocytes due to the fact that NADPH represents the only source of reducing power in red blood cells, where it is required to detoxify hydrogen peroxide among other compounds via glutathione antioxidant system [2].

G6PD deficiency is the most frequent enzymopathy in humans with an estimated global prevalence of 4.9% [3] and affects more than 400 million people around the world [2,4]. The G6PD deficiency causes severe disease ranging from mostly asymptomatic individuals to patients showing neonatal jaundice, acute episodes of hemolysis triggered by exogenous agents (acute infections, drugs or food), and chronic nonspherocytic hemolytic anemia [5].

This disease has genetic heterogeneity with around 186 mutations reported up to date, mainly point mutations that lead to single amino acid substitutions [6,7,8]. G6PD variants are generally classified according to their residual enzyme activity and hematology parameter of the patients, ranging from the most severe manifestations with less than 5% residual activity (Class I) to the mildest form (Class V). However, only around 10% of all recognized G6PD mutants have been characterized at the structural and functional level. Furthermore, from these 186 G6PD mutations, in Mexico the existence of 19 different mutants, including the mutants G6PD Zacatecas, Vanua-Lava and Viangchan, has been reported [9,10].

The Class I G6PD Zacatecas mutant involves substitution of guanine for thymine (G → T) at nucleotide (nt) 770 (exon 7) and was detected in a of 12-year-old boy from the state of Zacatecas, Mexico, which had antecedents of neonatal jaundice and hemolytic crisis during the first nine years of life requiring blood transfusion. This mutation results in the replacement of amino acid Arginine by Leucine 257 (R → L) [11], which in the tridimensional structure is located at a distance of ~9 Å from the substrate-binding site β-d-glucose-6-phosphate (G6P) (Figure 1B). G6PD Viangchan was first reported in a Laotian immigrant (from the city of Viangchan) of Calgary, Canada, and was characterized by severe enzyme deficiency [12]. G6PD Viangchan shows a G → A substitution in the nt 871 at exon 9, with a resultant change in the amino acid 291 (V → M), which has a distance of ~22 and ~25 Å from the substrate-binding site G6P and the structural NADP^+^ site, respectively (Figure 1B). This mutant is a Class II polymorphic Southeast Asian variant, associated with specific ethnic groups in tropical Asia [13,14,15,16]. However, the distribution of this G6PD mutation differs in each country; in Mexico, the polymorphic Southeast Asian variant is the third highest in frequency [11]. Finally, the Class II G6PD Vanua-Lava variant shows a T → C substitution at exon 5, nt 383, with a resultant change in the amino acid 128 (L → P) located at a distance of ~23 Å from the coenzyme site [17] (Figure 1B). The presence of G6PD Vanua-Lava variant in Mexico might be explained by population flow. There are historical antecedents about the arrival of slaves from several regions of Southeast Asia, departing from Manila and arriving to Acapulco during colonialism [9].

In this work, we report the construction, cloning, expression, detailed kinetic and thermostability studies of three purified clinical mutants of G6PD present in the Mexican population, G6PD Zacatecas (Class I), Vanua-Lava (Class II) and Viangchan (Class II), comparing them with the corresponding values of the human Wild Type (WT) G6PD enzyme. Finally, from the solved three-dimensional structure of the human G6PD protein, we define changes in the amino acid sequences that offer a molecular explanation of the effects of these mutations. Our results show that the mutations, regardless of the distance from the active site, affect the catalytic properties and structural parameters, and also change the three-dimensional structure which correlates with a more severe clinically phenotype.

## 2. Results and Discussion

### 2.1. Plasmid Construction and Heterologous Expression of Glucose-6-Phosphate Dehydrogenase (G6PD)

**Figure 2:**
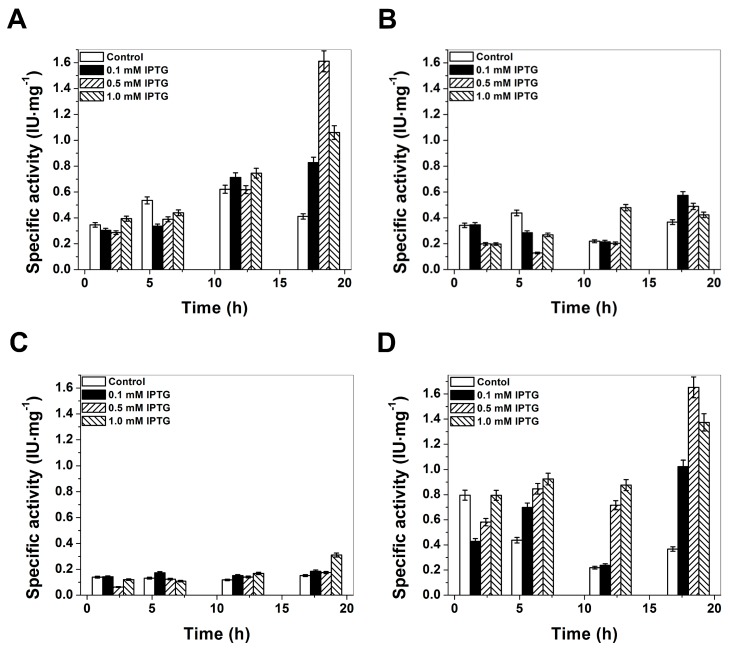
Heterologous expression of glucose-6-Phosphate Dehydrogenases (G6PDs) in *Escherichia coli* BL21(DE3)Δ*zwf*::*kan^r^*: (**A**) expression of human WT G6PD; (**B**) G6PD Zacatecas; and two Class II mutants; (**C**) G6PD Vanua-Lava and (**D**) G6PD Viangchan. Sonicated cell were centrifuged, and the resulting supernatants were used to measure the specific activity in each case. The G6PD specific activity was used as indicative of the expression levels of soluble recombinant protein. The standard deviations represent the value of triplicates samples.

In the present study, the overexpression of human G6PD enzymes in *Escherichia coli* (*E. coli*) BL21(DE3)Δ*zwf*::*kan^r^* cells was performed to produce large quantities of soluble recombinant human WT G6PD enzyme and three mutants (G6PD Zacatecas, Vanua-Lava and Viangchan). The specific activity was used as indicator of best overexpression conditions. As common strategy to attain large quantities of soluble recombinant human G6PD protein the growth temperature was reduced and the concentration of isopropyl-β-d-thiogalactopyranoside (IPTG) was modified. The reason is because, at low temperatures, the recombinant protein has more time to undergo proper folding. The expression was tested at 15, 25, and 37 °C, finding that soluble G6PD levels were most affected at 15 and 37 °C. Therefore, the best growth temperature for all mutants was at 25 °C. In all cases of the tested growth temperatures and IPTG induction, overexpression of G6PDs in *E. coli* largely resulted in the formation of inactive inclusion bodies. As shown in Figure 2, the best expression for the G6PD Zacatecas (R257L) mutant was obtained with a specific activity of 0.57 IU·mg^−1^ using 0.1 mM IPTG and 18 h of incubation (Figure 2B). For Class II G6PD Vanua-Lava (L128P) mutant the optimal expression conditions was obtained with 1 mM IPTG and 18 h of expression-time (specific activity of 0.30 IU·mg^−1^), while for Class II G6PD Viangchan (V291M) mutant, a higher expression of soluble protein was obtained with 0.5 mM and 18 h of incubation (specific activity of 1.6 IU·mg^−1^). It is interesting to note that, it was the first time that the specific activity obtained on the crude extract for one Class II G6PD mutant (Viangchan) is similar respect to WT G6PD enzyme. In all the G6PDs mutants previously biochemically characterized, even though the mutations were located in different parts of the three-dimensional structure of the WT G6PD protein, the expression of soluble protein measured by the specific activity was lower than WT G6PD (*i.e.*, 1.6 IU·mg^−1^; [19]) (Figure 2A). The level of overexpression of soluble recombinant human Class I G6PD Zacatecas and Class II G6PD Vanua-Lava are in concordance with the earlier results reported for Class I G6PD mutants (G6PD Nashville, Durham, Union) and Class II G6PD mutants (G6PD Valladolid, Union, Mexico city, Santa-Maria), where the specific activity in the crude extract was around 0.09 to 0.45 IU·mg^−1^ [20,21,22,23,24].

### 2.2. Purification and Characterization of Recombinant G6PDs

Purification of recombinant human G6PDs (WT and mutants) was accomplished by using a 2′,5′-ADP Sepharose 4B affinity column and also for additional chromatographic steps of anion exchange Q-Sepharose-4B column were required to improve the purity level (Figure S1A–D). Using anion exchange chromatography steps with salt gradient elution allowed to isolate the pure protein both of the WT G6PD and the three mutants. All the G6PD mutants eluted from Q-Sepharose-4B column were distributed in one main peak (Figure S1B–D).

SDS–PAGE analysis showed that G6PD derived from the WT (Figure S1A) and the mutants (Figure S1B–D) were obtained as a single band with greater than 96% purity, which allowed us to do the functional and structural trials for the mutants used in this work. A summary of the purification process is given in Table 1, which shows that 2 mg of WT G6PD protein is obtained from 1 L culture medium and for all the mutants the purification efficiency is lower. The yields from the purification of the G6PD Zacatecas, Vanua-Lava, and Viangchan are 40%, 42% and 43%, respectively. Although we observe that all the mutants have been expressed under optimal conditions, the G6PD mutants showed lower specific activity and yield compared to the WT G6PD [20,21,22,23,24]. The latter indicates that even though the mutations are located in different regions of the three-dimensional structure and that they are distant from the active site or the from structural NADP^+^ region, all exhibited a negative effect on the G6PD mutants expression. The lower yield is probably related to the degree of stability of the variants in the erythrocyte, which causes different clinical manifestations.

### 2.3. Functional Characterization

Steady-state kinetic experiments of recombinant human WT G6PD enzyme and the G6PD Zacatecas, Vanua-Lava, and Viangchan mutants were determined as previously reported [19,23,25]. All the mutants included in this study, Class I G6PD Zacatecas (58 s^−1^), Class II G6PD Vanua-Lava (142 s^−1^), and Class II G6PD Viangchan (145 s^−1^), showed lower catalytic constant (*k*_cat_) values when compared to WT G6PD (*i.e.*, 233 s^−1^) (Table 2). It is striking that the Class I G6PD Zacatecas mutant showed a dramatic decrease in catalytic efficiency of 75%, while both Class II G6PD Vanua-Lava and Viangchan mutants had a decrease in catalytic efficiency of 40%, respectively, in relation to the WT G6PD. However, the *K*_m_ values for the all mutants were higher for their two physiological substrates with respect to the WT G6PD enzyme. The Class I G6PD Zacatecas was the most affected mutant, which showed approximately three- and four-fold lower affinity (*K*mG6P = 111, *K*_m_NADP^+^ = 24 µM, respectively) for both physiological substrates compared to the WT G6PD enzyme (*i.e.*, *Km*·*G6P* = 38, *K*_m_NADP^+^ = 6 µM, respectively). These lower affinities are in concordance with earlier results reported by Kiani *et al.* [26], where the Zacatecas R257L mutation is close to the G6P binding regions (Figure 1B) as in the case of G6PD Wayne (R257G), affecting both the binding of G6P and NADP^+^ substrates in the active site. The alterations in the specific activity obtained for the proteins and the low purification yield for the G6PD mutants were consistent with the kinetic parameters of the purified enzymes. These data indicated that catalytic efficiency is affected differentially by these mutations and that the degree of damage in the specific activity depended on the location of the mutation in the tertiary structure (Figure 1B).

It is interesting to note that the kinetic parameters of the Class I G6PD Zacatecas mutant were in close agreement with earlier values for the Class I G6PD Nashville Union, Durham, where the catalytic constant (*k*_cat_) values showed a loss around 70% for all the mutants with respect to the WT G6PD enzyme [20,21,24]. However, despite of the loss of catalysis for these mutants, only the Class I G6PD Nashville and Durham showed a four-fold decrease in affinity for both physiological substrates [21,24], while both Class I G6PD Union and Yucatan showed a higher affinity for physiological substrates with respect to WT G6PD enzyme [21,23]. Finally, the Class II G6PD mutants (G6PD Vanua-Lava and Viangchan) characterized in this work showed moderate changes in kinetic parameters and were similar to the values previously reported for the Class II G6PD Wisconsin [21]. However, the *K*_m_ values obtained for the G6PD Viangchan mutant in this work differed of those, previously reported by Mang-Chiu *et al.* [27], where *K*_m_ values (*K*_m_G6P = 105, *K*_m_NADP^+^ = 12 µM) were obtained. Furthermore, simultaneously at our work, Boonyuen *et al.* [28] reported the *K*_m_ values of the recombinant G6PD Viangchan with His-tagged (*K*mG6P = 56, *K*_m_NADP^+^ = 34 µM), which are slightly different with those presented in this work.

### 2.4. Evaluation of Protein Stability

**Figure 3:**
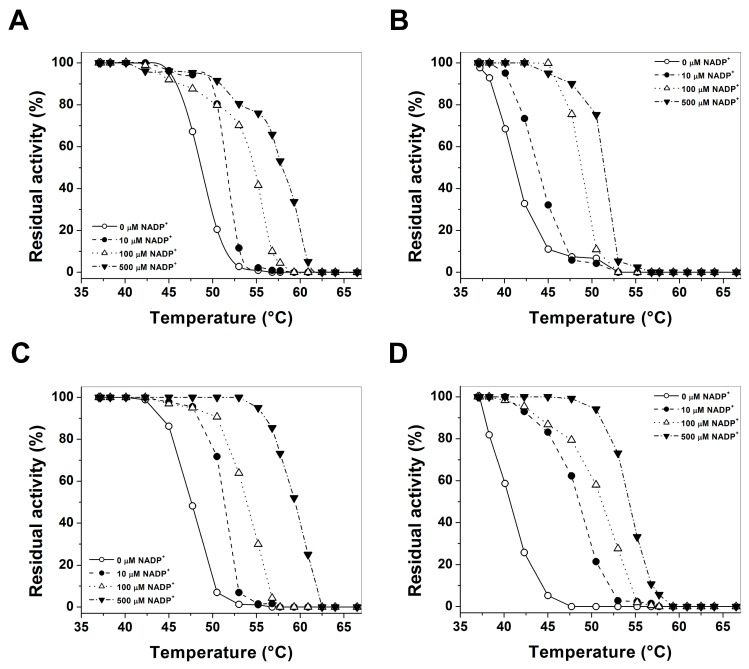
Thermostability assays of recombinant Wild Type glucose-6-phosphate dehydrogenase (WT G6PD) and the three mutants with different NADP^+^ concentrations: (**A**) WT G6PD; (**B**) G6PD Zacatecas (R257L); (**C**) G6PD Vanua-Lava (L128P); and (**D**) G6PD Viangchan (V291M). In all cases, 200 ng of total protein was used. Residual activity was expressed as a percentage of the activity for the same sample incubated at 37 °C. The assays were performed in duplicate; standard errors were lower than 5%. NADP^+^ concentrations: without NADP^+^ (○), 10 µM (•), 100 µM (∆) and 500 µM (▼) NADP^+^, respectively.

Thermostability is also a useful characteristic for assessing G6PD mutations [22,25,29,30], as it has been widely used to evaluate the stability of the active site of the G6PD enzymes, and the effect caused by the point mutation in catalytic efficiency. The thermostability of pure WT G6PD and these three pathological mutants were compared with different concentrations of NADP^+^ (0, 10, 100 and 500 µM, respectively). In Figure 3, we showed the *T*_50_ (temperature at which the enzyme loses 50% of its original activity after incubation for 20 min) values of WT G6PD enzyme and the mutants. In the absence of added NADP^+^ the *T*_50_ values were 48 and 47 °C for WT and Vanua-Lava enzymes, respectively; the *T*_50_ value for G6PD Zacatecas and G6PD Viangchan mutants was about 6 °C lower (41 °C) when compared with the obtained for WT G6PD (Figure 3A–D). When increasing concentrations of NADP^+^, the *T*_50_ values also increased for the three mutants (Figure 3A–D). The *T*_50_ values at 500 µM NADP^+^ for all the enzymes were 10 °C higher when compared with the values obtained without NADP^+^. It is interesting to note that the NADP^+^-dependent stabilization also has been observed in the G6PD Yucatan [23], Mahidol [22], Andalus [20], Plymouth [22] and Viangchan [27] mutants, even these mutations are not located near the active site or structural NADP^+^ binding in the native G6PD enzyme. However, in other Class I G6PD mutants, such as Nashville [21,23], Durham [23], Wisconsin [31], Fukaya [31] and Campinan [31], no protective effect was observed when NADP^+^ was increased because these mutations were located near to the dimer interface and structural NADP^+^ of the native G6PD enzyme. Besides, with all the concentrations of NADP^+^ the three mutants were less stable than the WT, suggesting that these enzymes have become less structurally stable and relaxed in the active site. The effect of these mutations probably does not affect the synthesis and folding of the protein in the erythrocytes, but the half-life could be reduced because the enzymes became unstable and loss catalytic efficiency.

**Figure 4:**
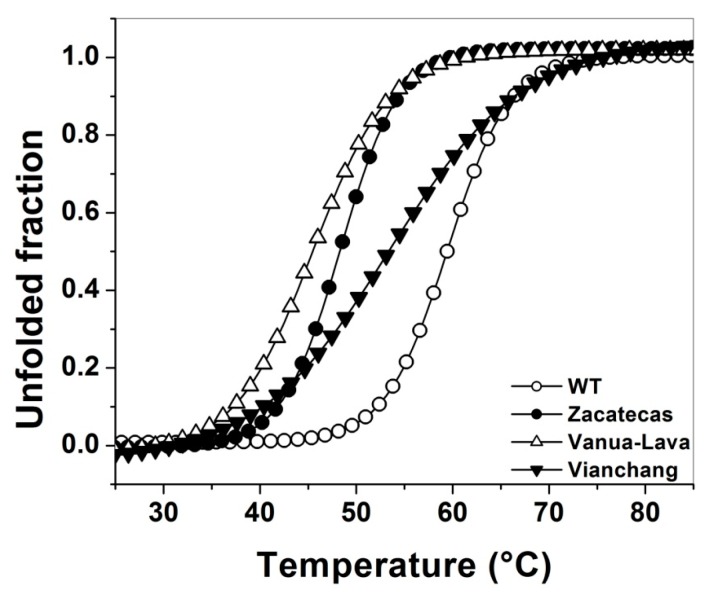
The mutations decrease the thermal stability of WT G6PD enzyme. The determination of melting temperature (*T*_m_) was monitored by recording the change in the CD signal at 222 nm when the temperature was increased progressively from 20 to 90 °C at 1°/2.5 min. In all cases, both the WT G6PD and the mutants were recorded at 0.8 mg/mL in 25 mM phosphate buffer pH 7.35. Experiments were performed in duplicate; standard errors were less than 5%.

We also characterize the structural stability of WT G6PD and mutants using thermal assays in which the half of the secondary structure is disrupted (*T*_m_) by temperature and structural changes are followed by circular dichroism (CD) signal at 222 nm (Figure 4). The effect of the mutations on the structural stability showed that the most affected enzymes were G6PD Zacatecas and Vanua-Lava mutants (loss of 13 and 11 °C in thermal stability, respectively). While the Class II G6PD Viangchan was least affected in the *T*_m_ value (loss of 6 °C) when compared with WT G6PD (*i.e.*, 59 °C). However, this last value in the *T*_m_ value are in agreement with earlier results for the Class I G6PD Nashville (R393H), Durham (K238R) and Yucatan (K429E) enzymes, where a decrease of 5 °C in thermal stability was obtained [21,23,24]. Furthermore, in two Class I G6PD clinical mutants, G6PD Fukaya (G488S) and G6PD Campinas (G488V) [31], where the mutations are in the vicinity of the “structural” NADP^+^ site, the *T*_m_ values are around to 10 °C lower respect to WT G6PD. These results confirm that the mutations in the G6PDs mutants have a strong effect in the stability of these structures that exhibiting significant differences at elevated temperatures. In addition, their effects on purification yield and the catalytic efficiency show that these mutants are the most severely affected proteins, while that WT G6PD enzyme remains stable at the global stability level, and that these changes may be related with alterations in the secondary, tertiary or quaternary structure, which could be the responsible of the different clinical manifestations.

**Figure 5:**
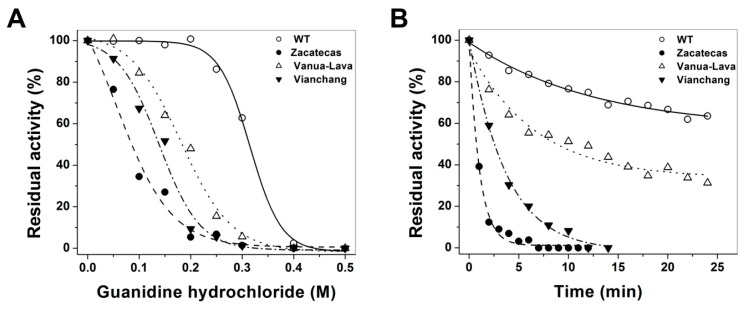
Stability of glucose-6-phosphate dehydrogenases (G6PDs) incubated with different concentrations of Gdn-HCl and inactivation assays. (**A**) Effects of Gdn-HCl on the activity of WT G6PD and the G6PD Zacatecas, Vanua-Lava and Viangchan mutants. All the enzymes were incubated at 0.2 mg/mL in 50 mM phosphate buffer pH 7.35 in the presence of the indicated concentrations of Gdn-HCl for 2 h at 37 °C; (**B**) Inactivation of WT G6PD and the mutants by 0.25 M at 37 °C. At the indicated times, aliquots were withdrawn from the samples and assayed for residual activity. In both assays, residual activity was expressed as a percentage of the activity for the same sample measured at 25 °C without Gdn-HCl and all enzymes were prepared and diluted immediately before used. Both assays were performed in duplicate; standard errors were less than 5%.

In order to further investigate the structural stability of the of recombinant human mutants enzymes induced by the mutations in the WT G6PD, inactivation assays by guanidine hydrochloride (Gdn-HCl) at different concentrations were performed for the WT G6PD and mutants. Residual activities of WT G6PD and mutants after 2 h of incubation at 37 °C with increasing concentrations of Gdn-HCl was used to determine the *C*_1/2_ values (Gdn-HCl concentrations at which the enzymes lose 50% of original activity in 2 h at 37 °C). As shown in Figure 5A, the WT G6PD was the most stable enzyme in the presence of Gdn-HCl. In contrast, the G6PD Zacatecas, Vanua-Lava and Viangchan mutants were more susceptible to Gdn-HCl, and they lost about 100% of its activity even after incubating with 0.3 M urea for 2 h. The *C*_1/2_ values of Gdn-HCl for the G6PD Zacatecas, Viangchan and Vanua-Lava mutants were 0.1, 0.15 and 2 M, respectively. Moreover, it is interesting to mention that when 50% of G6PD activity was lost in all the mutants, WT G6PD remained almost 100% of its original activity. These results suggest that mutants have a low conformational stability when compared with WT G6PD enzyme. Furthermore, based on the inactivation data, we decided to evaluated the lost of activity during the time-course inactivation with a fixed concentration of Gdn-HCl (0.25 M). The results indicate that for all G6PD mutants a single exponential decay of time-course inactivation was obtained (Figure 5B). The enzyme activity for Class I G6PD Zacatecas and Class II G6PD Viangchan decreased quickly, proving that both mutants were very susceptible to Gdn-HCl, and lost about 100% of its activity even after incubating with 0.25 M urea for only 10 min. These results are in agreement with the earlier date obtained in thermostability experiment, where both mutants were less stable and relaxed in the active site respect to WT G6PD enzyme. The Class II G6PD Vanua-Lava was the most resistant mutant to Gdn-HCl, because of the gradual loss of their activity (60%) over 30 min respect to WT G6PD enzyme that remained almost 75% of its original activity.

**Figure 6:**
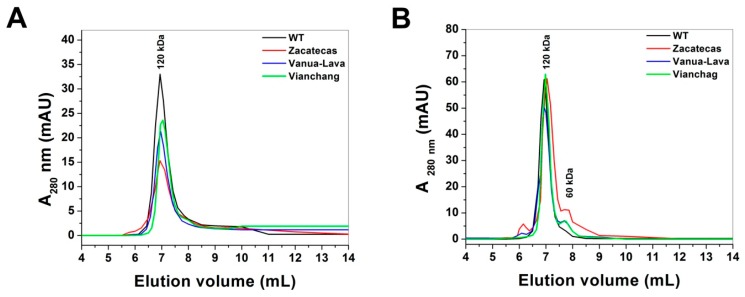
Gel filtration chromatography to evaluate the protein stability of glucose-6-phosphate dehydrogenases (G6PDs). The stability of native G6PDs dimers without (**A**) or with (**B**) Gdn-HCl were tested by FPLC, incubating the enzymes (0.2 mg/mL) at 37 °C for 2 h and then loading them onto a size-exclusion chromatography column. Thirty microliters samples of G6PDs protein solutions were loaded on Shodex Protein^®^ KW-802.5 column coupled to ÄKTA Primes FPLC system (Amersham Pharmacia Biotech, Piscataway, NJ, USA) previously equilibrated with 50 mM phosphate buffer at pH 7.35; flow rate: 1.0 mL/min.

Conformational changes in the quaternary structure of recombinant human G6PD Zacatecas, Vanua-Lava and Viangchan mutants were determined by analysis of the oligomeric protein state using Gel Filtration Chromatography (GFC). As shown in Figure 6A, both the WT G6PD and mutants elute as single peaks with retention volumes corresponding to native dimers (6.96 mL; 120 kDa), revealing that the loss of catalytic efficiency of 75% and 20% for the Class I G6PD Zacatecas and Class II G6PD Vanua-Lava, respectively, in relation to the WT G6PD is not due to dissociation of the native G6PD dimer.

To test the contribution of Gdn-HCl in the dimer stability and the loss of activity of the mutants, we performed GFC for the WT G6PD and the mutants to determine if the lost of activity in the mutants was due to native dimers dissociation of the protein or loss of local structure in the active site provoked by Gdn-HCl. As shown in Figure 6B, both the WT G6PD and the mutants elute as major single peaks with retention volumes corresponding to native dimers (6.96 mL, 120 kDa) and a lightly peak corresponding to the monomer form (7.8 mL; 60 kDa) was observed. The gel filtration elution profiles indicated that the loss of activity of G6PDs enzymes in the inactivation assays is not due to native dimer dissociation and that mutant enzymes are almost totally inactivated before its dissociation. Based on the aforementioned data, it can be suggested that in G6PDs mutants the loss of activity is due to local alterations on the tridimensional structure induced by the mutations and not native dimers dissociation.

### 2.5. Spectroscopic Characterization

**Figure 7:**
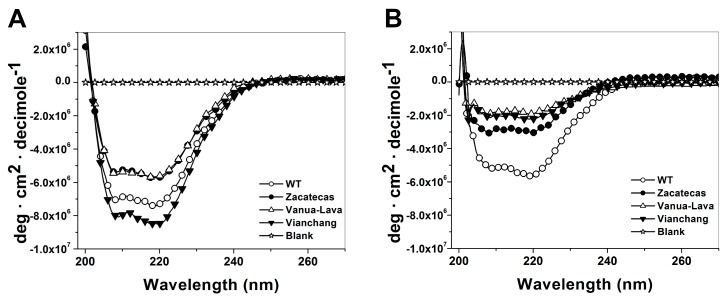
Spectroscopic characterization of recombinant human G6PD enzymes. Far-UV CD spectra of WT G6PD and mutants without (**A**) or with (**B**) 0.25 M Gdn-HCl were performed in spectropolarimeter (Jasco J-810^®^) equipped with a Peltier thermostatted cell holder; standard errors were less than 4%. In all cases, the protein concentration was 0.2 mg/mL and incubated by 2 h at 37 °C and after than measured by CD. For both trials, the spectra of blanks were subtracted from those that contained the protein.

Due to the studied G6PD mutants enzymes present low purification yield, diminished catalytic efficiency, they are also more thermolabile, with low conformational stability and are relaxed in the active site respect to WT G6PD enzyme, we decided to conduct spectroscopic analysis in order to characterize the structure of WT G6PD and the mutants by CD, fluorescence spectroscopy and their capacity to bind 8-anilinonaphthalene-1-sulfonate (ANS) assays. As shown in Figure 7A, the secondary structures of the WT G6PD and the three mutants of the protein in the far-UV region showed minimum absorption peaks at 208 and 220 nm (Figure 7A) that is consistent with the α-β structure of the protein previously reported by Au *et al.* [32]. The difference in lower pattern and intensity of the G6PD Zacatecas and Vanua-Lava indicate that both mutants lost secondary structure respect to WT G6PD enzyme.

Furthermore, in order to explore if the structural changes evaluated by activity loss of the mutants during the Gdn-HCl time-course inactivation assays was due to a wider structural disruption or a local effect, we decided to evaluate the secondary structures both of the WT G6PD and the three mutants in presence of Gdn-HCl (0.25 M). As shown in Figure 7B, the results showed that all G6PD mutants tested lost secondary structures. The most susceptible mutants were the G6PD Viangchan, Vanua-Lava and Zacatecas, with a loss of secondary structure of 76%, 64% and 43% respectively, compared with themselves without Gdn-HCl. As expected the WT G6PD was the most resistant enzyme to Gdn-HCl, losing near to 18% of secondary structure. These results indicates that the loss of activity observed both in the inactivation and time-course inactivation assays for G6PD Zacatecas and Viangchan mutants were due to loss of local structure and not the dimer dissociation. Probably, this loss of secondary structure caused changes to its native conformation near the active site and the protein could be degraded causing clinical manifestations as previously described.

**Figure 8:**
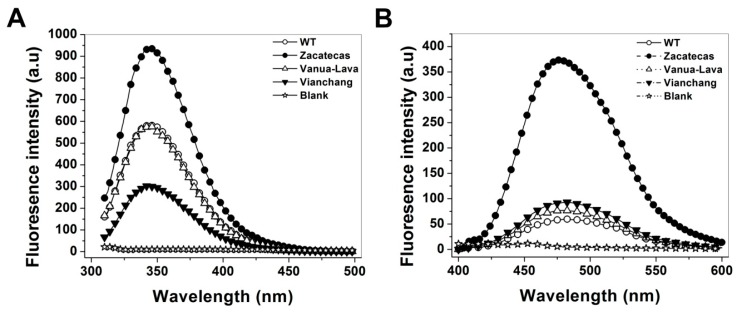
Conformational changes in the tertiary structure of recombinant human WT G6PD enzymes and mutants. (**A**) Fluorescence emission spectra were performed in a Perkin-Elmer LS-55 fluorescence spectrometer. The assays were conducted with a protein concentration of 0.1 mg/mL; (**B**) ANS fluorescence spectra were obtained using an excitation wavelength of 395 nm and recording emission spectra from 400 to 600 nm. Values obtained from buffer containing ANS without protein (open stars) were subtracted from the recordings with protein. The experimental conditions for all the experiments are described in the Materials and Methods Section.

**Figure 9:**
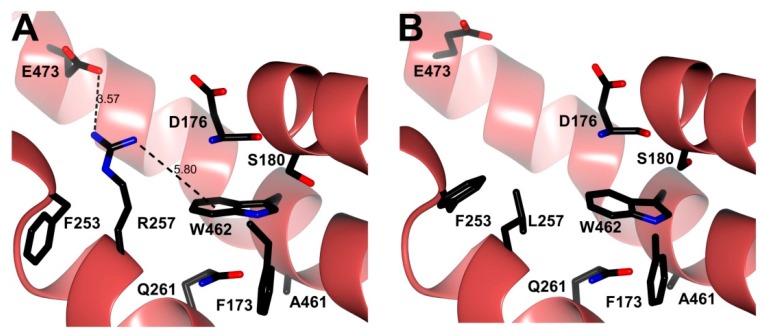

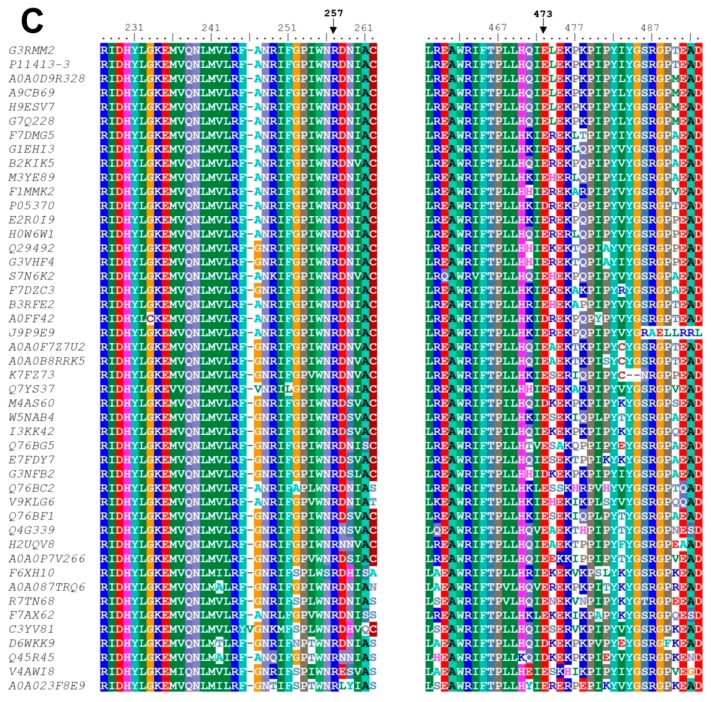
Structural comparison between the human WT G6PD enzyme and the Class I G6PD Zacatecas mutant. (**A**) Crystallographic structure of human WT-G6PD enzyme (pale crimson) (PDB entry 2BH9); (**B**) Minimized model of the Class I G6PD Zacatecas variant (pale crimson) with the *in silico* R257L mutation. Note that the R257 (black cylinders) residue forms a weak cation–π interaction with W462 and a salt bridge with E473 in (**A**), and their absence in the *in silico* R257L mutant in (**B**). Distances are in Å; (**C**) Alignment of amino acid sequence of G6PD with homologs from different species from *Gorilla gorilla gorilla* (G3RMM2), *Homo sapiens* (P11413-3), *Chlorocebus sabaeus* (A0A0D9R328), *Papio anubis* (A9CB69), *Macaca mulatta* (H9ESV7), *Macaca fascicularis* (G7Q228), *Equus caballus* (F7DMG5), *Camelus dromedarius* (G1EHI3), *Rhinolophus ferrumequinum* (B2KIK5), *Mustela putorius furo* (M3YE89), *Bos taurus* (F1MMK2), *Rattus norvegicus* (P05370), *Canis lupus familiaris* (E2R0I9), *Cavia porcellus* (H0W6W1), *Macropus robustus* (Q29492), Sarcophilus *harrisii* (G3VHF4), *Myotis brandtii* (*S7N6K2*), *Ornithorhynchus anatinus* (F7DZC3), *Sorex araneus* (B3RFE2), *Mus caroli* (A0FF42), *Canis lupus familiaris* (J9P9E9), *Crotalus adamanteus* (A0A0F7Z7U2), *Pelodiscus sinensis* (K7FZ73), *Bos indicus* (Q7YS37), *Xiphophorus maculatus* (M4AS60), *Lepisosteus oculatus* (W5NAB4), *Oreochromis niloticus* (I3KK42), *Ambystoma mexicanum* (Q76BG5), *Danio rerio* (E7FDY7), *Gasterosteus aculeatus* (G3NFB2), *Cephaloscyllium umbratile* (Q76BC2), *Callorhinchus mili* (V9KLG6), *Lepisosteus osseus* (Q76BF1), *Rhabdosargus sarba* (Q4G339), *Takifugu rubripes* (H2UQV8), *Scleropages formosus* (A0A0P7V266), *Xenopus tropicalis* (F6XH10), *Stegodyphus mimosarum* (A0A087TRQ6), *Capitella teleta* (R7TN68), *Ciona intestinalis* (F7AX62), *Branchiostoma floridae* (C3YV81), *Tribolium castaneum* (D6WKK9), *Rhipicephalus microplus* (Q45R45), *Lottia gigantea* (V4AWI8), *Triatoma infestans* (A0A023F8E9), *Anopheles gambiae* (H2KMF3), *Apis mellifera* (A0A023FG14), *Musca domestica* (T1PD22), *Aedes aegypti* (Q0IEL8) performed with BioEdit V.7.2.5. The uniform colors indicate conserved amino acid in the sequences reported. Colorless represent non-conserved amino acid sequences. The arrows indicate the position R257 and E473 residues are highly conserved in different organisms.

The presence of 7 tryptophan residues/monomer in the human G6PD was used to evaluate the structural alterations in the three-dimensional structure of the proteins [32]. Furthermore, fluorescence emission maxima have been served to monitor modifications in the microenvironment of the tryptophan residues. As shown in Figure 8A, the fluorescent intensity for the Class I G6PD Zacatecas enzyme increased two-fold; while, the fluorescence intensity of G6PD Vanua-Lava was the same respect to WT G6PD. The increase on the intrinsic fluorescence intensity for the Class I G6PD Zacatecas is in concordance with the earlier results for the Class I G6PD Durham [24], where the intrinsic fluorescence intensity was increased two-fold. This increase of intrinsic fluorescence intensity for the Class I G6PD Zacatecas suggests that the mutation R257L probably causes modifications in the microenvironment of the W462 residue, causing the exposure of this tryptophan to a more hydrophilic environment in the three-dimensional structure of this protein (Figure 9A). In fact, the R257 forms a weak cation–π interaction with W462 (Figure 9A), which is lost in the R257L mutant (Figure 9B). The changes observed in intrinsic fluorescence intensity were corroborated by ANS assays.

To determine the possible degree of structural perturbation, both the WT G6PD and the three G6PD mutants, the emission fluorescence spectra of ANS was monitored. ANS assays have been widely used to monitor conformational changes of enzyme and their fluorescent molecular probes indicate a full exposure of hydrophobic sites; when proteins contain more hydrophobic regions, they will exhibit higher ANS fluorescence signals [23,24,33]. The results obtained indicate that the native WT G6PD enzyme has a maximal fluorescence emission spectrum of 60 arbitrary units (AU) at 482 nm (Figure 8B). The G6PD Vanua-Lava and Viangchan mutants produces an ANS spectrum of 76 and 92 AU, respectively, which corresponds to an increase of 26% fluorescence intensity respect to WT G6PD while the emission spectrum for Class I G6PD Zacatecas showed maximal fluorescence of 373 AU at 478 nm, which corresponds to an increase of 700% fluorescence intensity (Figure 8B). The increase in the fluorescence intensity for the Class I G6PD Zacatecas more than 700% suggest that the point-mutation in the position 257 of Arginine by Leucine induce the exposure of buried hydrophobic regions on the mutant enzyme. In addition to the loss of the cation–π interaction between R257 and W462, the exposure of a hydrophobic region in the Class I G6PD mutant is also probably due to the loss of a salt bridge between R257 and E473 (Figure 9A) in the R257L mutant (Figure 9B). These results suggest that the three-dimensional structure of this mutant is to some extent open, and presents loss of the secondary structure and exposure of hydrophobic regions (Figure 9B). Furthermore, it is interesting to mention that both R257 and E473 residues are highly conserved in different organisms as shown in the alignment with 50 G6PDs sequence of different organism (Figure 9C). These changes in the ANS fluorescence are in accordance with the previous data reported for the G6PD Nashville, Wisconsin, Mexico City, Valladolid and Durham mutants where an increased has been observed around of 1.8- to 2.6-fold higher respect to WT G6PD enzyme [21,23,24]. Furthermore, these data, together with the low purification yield, diminished catalytic efficiency and the thermostability of the mutated proteins, reinforce the idea that G6PD mutants have an unstable structure with low conformational stability and are relaxed in the active site respect to WT G6PD enzyme and that the degree of damage seems depend on the location of the mutation in the tertiary structure domains.

## 3. Materials and Methods

### 3.1. Gene Synthesis and Plasmid Construction

The recombinant plasmid pET-HisTEVP-*g6pd* [19] containing the full human *g6pd* gene (NM_001042351 access) was used as a template to generate by site-directed mutagenesis of the desired mutations: G6PD Zacatecas (R257L), Vanua-Lava (L128P), and Viangchan (V291M) (Table 3). Moreover, two flanking primers (Table 3) were designed for PCR amplification of the human *g6pd* gene containing *Nde*I and *Bpu11021* restriction sites (underlined) at the 5′ and 3′ ends, respectively. Furthermore, internal forward and reverse sequencing primers were used to verify the sequence for each mutant used in this study according to Gomez-Manzo *et al.* [23].

The constructions of *g6pd* mutants were performed by PCR; all PCR products were obtained using a Mastercycler gradient thermal cycler from Eppendorf (Hamburg, Germany). The desired mutations were obtained by overlapping the products derived from the first and second PCR rounds, as previously described [23] by flanking *NdeI* forward and *Bpu11021* reverse primers, which in turn contained the desired restriction sites. PCR conditions were the same employed by Gómez-Manzo *et al.* [19,23].

All PCR products for each mutant were analyzed by 1% agarose gel electrophoresis and amplicons of the expected size (1545 bp) were purified with the QIAquick Gel Extraction Kit (QIAGEN) (Valencia, CA, USA). The purified PCR products were ligated into the pJET 1.2 vector (CloneJET PCR Cloning Kit; Thermo Scientific, Hudson, NH, USA) and each construction was transformed into competent *E. coli* TOP-10 cells, which were grown at 37 °C overnight on Luria Bertani (LB)-agar plate supplemented with 100 µg/mL ampicillin (*Amp^R^*). To confirm the fidelity and desired mutation in the *g6pd* gene sequence, plasmid DNA of each mutant was isolated and full sequenced. The pJET 1.2 vector containing the verified sequence for each mutant *g6pd* gene was digested with restriction enzymes *Nde*I and *Bpu11021*, and sub-cloned into the pET-3a plasmid (Novagen, Madison, WI, USA). The recombinant expression plasmids with the desired mutations were identified as follows: pETgR257L, pETgL128P and pETgV291M (Table 3). They were then transformed into competent *E. coli* BL21(DE3)Δ*zwf*::*kan^r^* [23] that were grown at 37 °C overnight on an LB-agar plate containing 100 µg/mL ampicillin (*Amp^R^*) and 100 µg/mL kanamycin (*Kan^R^*) (both antibiotics).

### 3.2. Expression and Purification

Recombinant G6PD Zacatecas, Vanua-Lava, and Viangchan mutants were expressed in *E. coli* BL21(DE3)Δ*zwf*::*kan^r^*. As previously described by Gómez-Manzo *et al.* [23,24], the optimal expression conditions of G6PD soluble protein, was performed on 50 mL Luria Bertani culture medium using three expression temperatures (15, 25, and 37 °C, respectively) and three IPTG (isopropyl-β-d-thiogalactopyranoside) concentrations (0.1, 0.5, and 1 mM, respectively), and conducted for 2, 6, 12 and 18 h time courses. At the indicated time points, the cells were concentrated by centrifugation at 5000 rpm and 4 °C for 10 min, harvested, resuspended in lysis buffer [23,24], and broken down by sonication. The crude extract was clarified by centrifugation and aliquots from the supernatant were used to calculate specific G6PD activity.

The best conditions for protein expression (temperature, IPTG concentration and expression time) for each G6PD mutant was used to grow 2 L of LB medium containing 100 µg/mL of *Amp^R^* and *Kan^R^*. The cells were centrifuged, resuspended in lysis buffer, and disrupted by sonication. The crude extract suspension was centrifuged at 23,000× *g* for 30 min at 4 °C to remove the cell debris, and the supernatant was used for purifying the respective enzymes.

Purification of recombinant human G6PDs (WT and mutants) was accomplished with two differential ammonium sulfate precipitation (from 0% to 25% and the resulting supernatants were precipitated at 55%). Then, the pellet was dissolved in equilibrium buffer (150 mM NaCl plus 50 mM Na_2_PO_4_, pH 7.35), applied to a 2′,5′-ADP Sepharose 4B affinity and anion exchange Q-Sepharose-4B columns (Sigma-Aldrich, St. Louis, MO, USA) as reported for WT G6PD with minor modifications [23,24]. Fractions showing G6PD activity were pooled and concentrated with Amicon YM-30 tubes (Millipore Corp., Bedford, MA, USA). The NaCl was removed from the sample by 5 successive steps of ten-fold dilution in 0.025 M phosphate buffer, pH 7.4. Finally, the purity of the recombinant enzymes was confirmed on SDS-PAGE (12%) gels stained with Coomassie brilliant blue (R-250) (Sigma-Aldrich).

### 3.3. Functional Characterization

Steady-state kinetic experiments of WT G6PD and the mutants were determined spectrophotometrically at 25 °C by monitoring the reduction of NADP^+^ at 340 nm, as previously reported [19,23,25]. Standard reaction mixture contained 100 mM Tris-HCl buffer at pH 8.0, plus 3 mM MgCl_2_, 1 mM of glucose-6-phosphate and 1 mM NADP^+^, respectively. One unit (U) of G6PD activity is the amount of enzyme required to produce 1 µmol of NADPH per minute under the assay conditions, while the specific activity was defined as units per mg of protein. The reaction was initiated with the addition of 200 ng of each G6PD enzyme. Initial velocity data were obtained by varying one substrate (2.5 to 200 µM), while the second substrate was fixed at saturating concentration. The *k*_cat_ for each variant was calculated according to previous reports [19,23].

### 3.4. Evaluation of Protein Stability

For thermal inactivation experiments, the recombinant human WT G6PD enzyme and the mutants freed from loosely bound NADP^+^ obtained by serial dilution using Amicon YM-30 tubes (Millipore Corp.) were diluted at a final enzyme concentration of 200 µg/µL in 50 mM potassium phosphate buffer pH 7.35 containing 2 mM MgCl_2_ and different concentrations of NADP^+^ (0, 10, 100 and 500 µM) as previously reported [19,23]. After incubating for 20 min at different temperatures ranging from 37 to 61 °C, the samples were cooled and the residual enzyme activity was measured in the standard reaction mixture at 25 °C. The residual activity was expressed as a percentage of the activity of the same enzyme incubated at 25 °C. All the thermal inactivation tests were repeated at least three times.

The thermal stability and unfolding of the recombinant human WT G6PD enzyme and mutants were followed by CD using a spectropolarimeter (Jasco J-810^®^) as previously described [19,23]. The protein thermal unfolding at 0.8 mg/mL in phosphate buffer was measured as the change in the CD signal at 222 nm in temperature scans ranging from 20 to 90 °C at a rate of 1 °C/2.5 min increases. The spectra of blanks were subtracted from those that contained the recombinant human WT G6PD enzyme and mutants, respectively. The fraction at which 50% of the protein is folded and unfolded is expressed as the melting temperature (*T*_m_) value was calculated as previously reported [19,23].

Stability in the presence or absence of Gdn-HCl was performed to evaluate the changes induced by the mutations in the WT G6PD enzyme. Purified WT G6PD and mutants, freed from loosely bound NADP^+^ as described above, were adjusted at 0.2 mg/mL protein concentration and Gdn-HCl was added back to render concentrations of 0.05, 0.10, 0.15, 0.20, 0.25, 0.30, 0.40 and 0.5 M, respectively. All samples were incubated at physiological temperature (37 °C) for 2 h and subsequently residual activity was measured. Residual activity was expressed as a percentage of the activity for the same sample measured at 25 °C without Gdn-HCl. Furthermore, time-course inactivation of purified WT G6PD and mutants was performed at 0.2 mg/mL protein concentration and 0.25 M of Gdn-HCl in 50 mM phosphate buffer pH 7.35 at 37 °C. Aliquots from these incubations at different time points were withdrawn, and residual activity was measured at intervals over a period of up to 30 min. Data are reported as the percentage of residual activity, using the activity of each enzyme incubated without Gdn-HCl as 100% activity. All enzymes were prepared and diluted immediately before use.

Another way to detect conformational changes in the tertiary structure of recombinant human G6PD mutants was determining by the oligomeric state of the protein by GFC. Furthermore, we performed this same assay in presence of Gdn-HCl to determine if the effect of Gdn-HCl in the specific activity was due to native dimer dissociation of the G6PD or loss of structure near the active site. Both assays were conducted with a protein concentration of 0.2 mg/mL in 50 mM phosphate buffer at pH 7.35. The concentrated proteins were then applied to a Shodex Protein^®^ KW-802.5 column coupled to ÄKTA Primes FPLC system (Amersham Pharmacia Biotech) and eluted with the same buffer at a flow rate of 0.5 mL/min. The column was calibrated with gel filtration standard from Biorad with molecular weight markers ranging from 1350 to 670,000 Daltons.

### 3.5. Spectroscopic Characterization

Analysis of secondary structure of the recombinant human WT G6PD and the mutants enzymes was analyzed by CD in a spectropolarimeter (Jasco J-810^®^) equipped with a peltier thermostated cell holder [19,23,24]. Far-UV CD spectral scans at 25 °C were performed from 200 to 260 nm at 1 nm intervals in a quartz cell with a path length of 0.1 cm. The assays were conducted with a protein concentration of 0.2 mg/mL in 50 mM phosphate buffer at pH 7.35. Furthermore, CD measurements of recombinant human WT G6PD and the mutant’s enzymes were performed with 0.25 M of Gdn-HCl to evaluate if the loss activity in the time-curse inactivation assay was due to a wider structural disruption or local effect in the secondary structure. For both trials, the spectra of blanks were subtracted from those that contained the protein.

Analysis of conformational changes in the tertiary structure of recombinant human WT G6PD enzymes and mutants were evaluated by intrinsic fluorescence and their capacity to bind 8-anilinonaphthalene-1-sulfonate (ANS) assays. Both assays were performed in a Perkin-Elmer LS-55 fluorescence spectrometer (Perkin Elmer, Wellesley, MA, USA) as formerly reported [19,23]. The fluorescence emission spectra from 310 to 500 nm were recorded after excitation at 295 nm, with an excitation and emission slits of 4.5 and 3.7 nm, respectively. All the assays of intrinsic fluorescence were conducted with a protein concentration of 0.1 mg/mL. ANS assays were performed in 25 mM phosphate buffer, pH 7.4 at 25 °C, using an excitation wavelength of 395 nm and recording emission spectra from 400 to 600 nm with an excitation and emission slits of 10 and 10 nm, respectively. The final concentrations of ANS and the recombinant human G6PD enzymes were the same as previously reported [19,23]. For both spectroscopic assays, the spectra of blanks without protein were subtracted from the experimental samples that contained the respective protein.

### 3.6. In Silico Mutagenesis and Modeling

Mutations in the crystallographic structure of the human WT-G6PD (PDB entry 2BH9) at positions 128, 257 and 291 were generated *in silico* using the standard rotamer library of Coot [35]. The mutant models were subjected to energy minimization using YASARA software [36]. The graphical representations were also constructed with CCP4mg version 2.10.4 [18].

## 4. Conclusions

The biochemical results obtained in this study of the three recombinant G6PD mutants, Zacatecas (R257L), Vanua-Lava (L128P), and Viangchan (V291M), showed a low purification yield of G6PD mutants and are effect in their kinetic parameters, where the G6PD Zacatecas was the most affected mutant showing three- and four-fold lower affinity for both physiological substrates compared with the WT G6PD enzyme. The structural characterization suggests that the mutations in the WT G6PD have a strong effect on the global stability of G6PD. In addition, these mutants have become less structurally stable with low conformational stability and are relaxed in the active site. Computational analyses based on solved three-dimensional structure of the human G6PD protein offers a molecular explanation of the G6PD Zacatecas mutant. Sequence alignments show that R257 and E473 in G6PD are highly conserved and form a salt bridge, implying its importance in G6PD structure and function. In G6PD Zacatecas mutant (R257L), a salt bridge of R257 with E473 amino acid is lost, which could be critical for both catalytic activity and overall protein stability that correlate with a more severe phenotype. Finally, although all the mutations are distant from the active site, they affect the functional and structural parameters indicating that these mutants are probably synthesized and folded correctly in the red cells, these mutant proteins are particularly susceptible to proteolysis or other damage in the half-life of red blood cells, and consequently responsible for the different clinical manifestations. Finally, in view of the functional and structural alterations in the G6PD mutants, we could go in search of a synthetic compound or a molecule that stabilizes at the mutants and prevents the different clinical manifestations, improving the enzyme function and also the life quality of patients.

## Figures and Tables

**Table 1:** Purification summary of all recombinant human glucose-6-phosphate dehydrogenase (G6PD) enzymes.

G6PD Enzyme	Total Protein (mg)	Specific Activity (IU·mg^−1^)	Total Activity (IU)	Yield (%)
Wild Type	2.1	230	483	61
Zacatecas	1.46	58	85	40
Vanua-Lava	0.5	182	91	42
Viangchan	1.32	228	300	43

Values are for a typical expression purification experiment. The specific activity was measured under a standard procedure described in Materials and Methods. The result varies <10% from batch to batch.

**Table 2:** Catalytic properties of human G6PD and the mutants.

Kinetic Constants	WT-G6PD	Mutants		
		Zacatecas	Vanua-Lava	Viangchan
*K*mG6P (µM)	38.49	111	34	42
*K*_m_NADP^+^ (µM)	6.16	24	18	17
*k*_cat_ (s^−1^)	230	58	142	145
*k*_cat_/*K*_m_·G6P (s^−1^·M^−1^)	5.97 × 10^6^	0.52 × 10^6^	4.17 × 10^6^	3.45 × 10^6^
*k*_cat_/*K*_m_NADP^+^ (s^−1^·M^−1^)	37.33 × 10^6^	2.41 × 10^6^	7.88 × 10^6^	8.52 × 10^6^

The kinetic constants estimated for the WT G6PD and the Class I G6PD Zacatecas and two Classes II G6PD Vanua-Lava and Viangchan mutants. The parameters in each case were obtained from three independent experiments and from different enzyme preparations.

**Table 3:** Strains, plasmids, and primers used in this study.

Strain *E. coli*	Relevant Characteristic(s) or Sequence	Source and/or Reference
BW25113	F^−^, DE(araD-araB)567, lacZ4787(del)::rrnB-3, LAM^−^, rph-1, DE(rhaD-rhaB)568, hsdR514	[34]
BL21(DE3)Δ*zwf*::*kan^r^*	F^−^ ompT gal dcm lon hsdS_B_(r_B_^−^ m_B_^−^) λ(DE3 (lacI lacUV5-T7 gene 1 ind1 sam7 nin5)) Δ*zwf-777*::*kan*	[23]
**Plasmids**		
pET*g6pd*	pET-3a carrying the human *g6pd* gene, *Amp^R^*	[23]
pETgR257L	pET-3a carrying the human *g6pd* gene with a R257L mutation in the G6PD protein, *Amp^R^*	This study
pETgL128P	pET-3a carrying the human *g6pd* gene with an L128P mutation in the G6PD protein, *Amp^R^*	This study
pETgV291M	pET-3a carrying the human *g6pd* gene with an V291M mutation in the G6PD protein, *Amp^R^*	This study
**Mutagenesis**	**Primer Sequence**	
R257L fw	5′-GGATCATCC**T**GGACGTGATG-3′	This study
R257L rev	5′-CATCACGTCC**A**GGATGATTCC-3′	This study
L128P fw	5′-GAATGCCC**T**CCACCTGGG-3′	This study
L128P rev	5′-CTTACGGG**A**GGTGGACCC-3′	This study
V291M fw	5′-GAGAAGGTCAAG**A**TGTTGAAATG-3′	This study
V291M rev	5′-CATTTCAACA**T**CTTCACCTTCTC-3′	This study

The locations of the mutagenic oligonucleotides are in bold.

## Supplementary Materials

Supplementary materials can be found at http://www.mdpi.com/1422-0067/17/5/787/s1.
