# Supplementary material for: Functional and Biochemical Characterization of Three Recombinant Human Glucose-6-Phosphate Dehydrogenase Mutants: Zacatecas, Vanua-Lava and Viangchan
Source: Int J Mol Sci. 2016 May 21;17(5):787. doi: 10.3390/ijms17050787 (PMC4881603; doi:10.3390/ijms17050787)
Supplement: Supplementary file 1 [file ijms-17-00787-s001.pdf]

Supplementary Materials: Functional and Biochemical Characterization of Three Recombinant Human Glucose-6-Phosphate Dehydrogenase Mutants: Zacatecas, Vanua-Lava and Viangchan

Saúl Gómez-Manzo, Jaime Marcial-Quino, America Vanoye-Carlo, Hugo Serrano-Posada, Abigail González-Valdez, Víctor Martínez-Rosas, Beatriz Hernández-Ochoa, Edgar Sierra-Palacios, Rosa Angélica Castillo-Rodríguez, Miguel Cuevas-Cruz, Eduardo Rodríguez-Bustamante and Roberto Arreguin-Espinosa

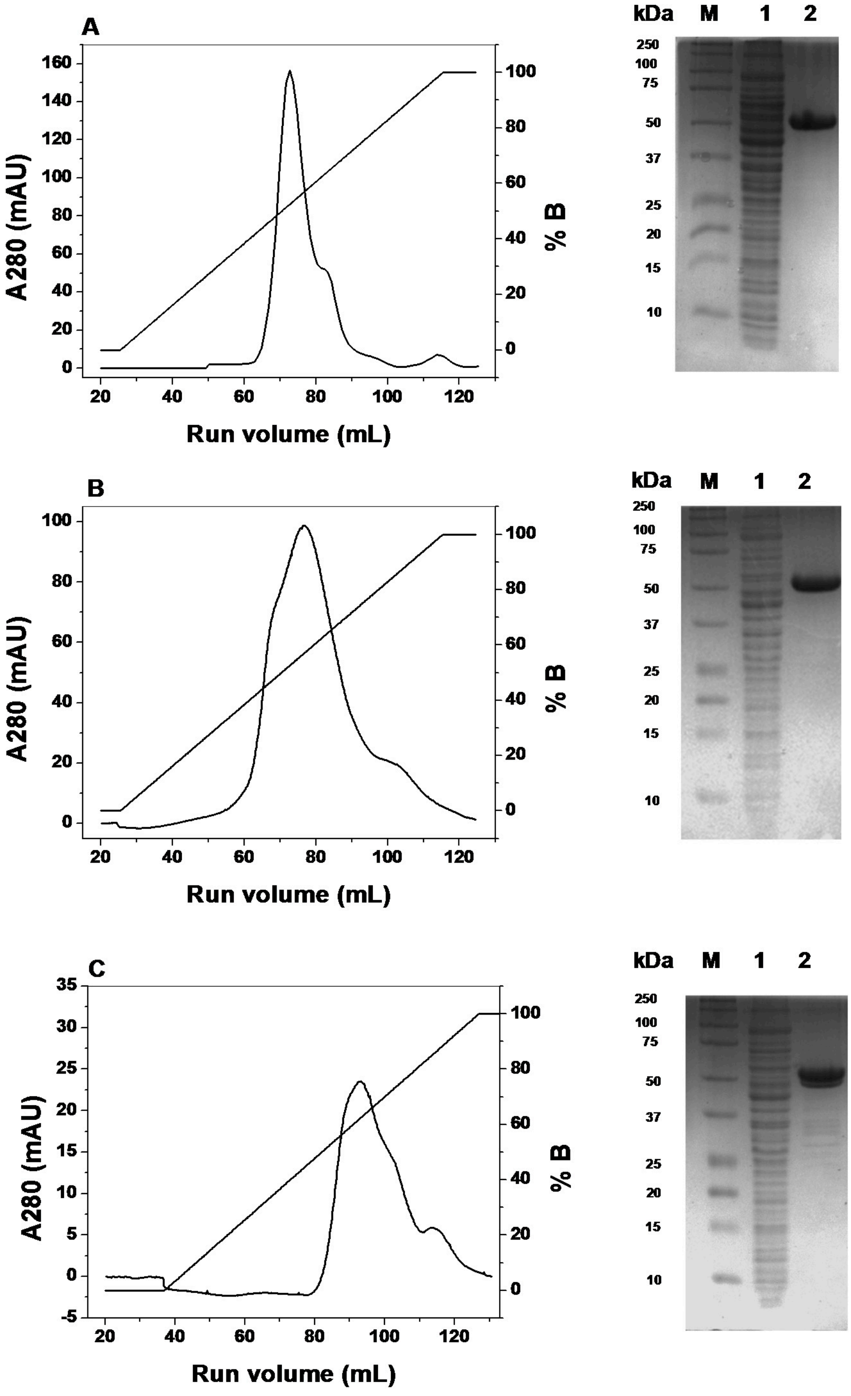

Figure S1. *Cont.*

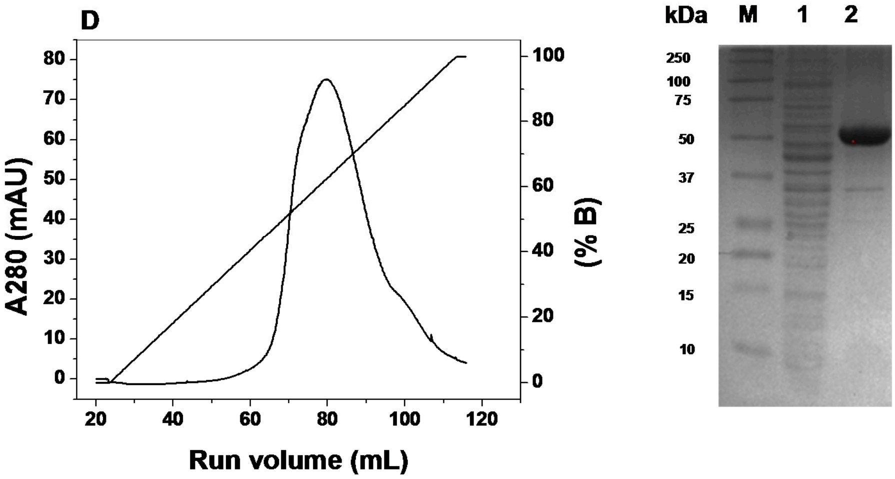

Figure S1. Purification and sodium dodecyl sulfate-polyacrylamide gel electrophoresis (SDS-PAGE) of the recombinant human glucose-6-phosphate dehydrogenase (G6PD) enzymes. Chromatogram from purification (**A**) Wild Type glucose-6-phosphate dehydrogenase (WTG6PD); (**B**) G6PD Zacatecas; (**C**) G6PD Vanua-Lava and (**D**) G6PD Viangchan by cation exchange columns using the ÄKTA Prime Plus system (Piscataway, NJ, USA). Protein loading, the column was washed with 5-bed column volumes in 50 mM phosphate buffer at pH 7.35; flow rate: 2.0 mL/min. The proteins were eluted by a linear concentration (0–0.35 M) gradient of NaCl in the starting buffer. SDS-PAGE analysis of purified enzymes. M: molecular weight marker Broad Range SDS-PAGE standards from Biorad; Lane 1, crude extract; Lane 2, eluted fraction. Each lane was loaded with 10 μg of protein and stained by Coomassie brilliant blue R-250.
